# Research on truck-drone collaborative route planning for rural logistics delivery services

**DOI:** 10.1038/s41598-024-83149-1

**Published:** 2024-12-30

**Authors:** Yong Wang, Suo Yang, Xi Vincent Wang, Lihui Wang

**Affiliations:** 1 School of Management, Wuhan University of Science and Technology, Wuhan, 430080 China; 2 Department of Production Engineering, KTH Royal Institute of Technology, 11428 Stockholm, Sweden

**Keywords:** Rural logistics, Trucks, Drones, Collaborative path planning, Environmental social sciences, Energy science and technology, Mathematics and computing

## Abstract

This study investigates the implementation of collaborative route planning between trucks and drones within rural logistics to improve distribution efficiency and service quality. The paper commences with an analysis of the unique characteristics and challenges inherent in rural logistics, emphasizing the limitations of traditional methods while highlighting the advantages of integrating truck and drone technologies. It proceeds to review the current state of development for these two technologies and presents case studies that illustrate their application in rural logistics. Building on this analysis, a collaborative path planning method is proposed, establishing a path optimization model and designing an enhanced simulated annealing algorithm. The effectiveness of this approach is validated through simulation experiments, which reveal that the collaborative delivery system for trucks and drones can significantly boost efficiency, lower costs, and improve service quality. In conclusion, the research findings and potential future research directions are discussed to offer theoretical insights and practical guidance for further innovations in rural logistics technology.

**Supplementary Information:**

The online version contains supplementary material available at 10.1038/s41598-024-83149-1.

With the rapid development of China’s rural economy and the change in consumption structure, the demand for logistics services in rural areas is increasing, especially regarding the distribution of daily necessities and e-commerce goods. Nevertheless, the conventional logistics distribution model faces numerous challenges, such as low efficiency, high costs, and a limited service range. This is particularly true in remote and mountainous regions with complicated road conditions, leading to elevated logistics distribution costs.

In recent years, the combination of truck and drone technology has attracted considerable attention as a promising solution to the challenges of rural logistics distribution. With their robust adaptability and substantial carrying capacity, trucks can transport large volumes of goods while simultaneously lowering operating costs and enhancing distribution efficiency. On the other hand, the rapid advancements in drone technology present vast application opportunities in logistics, particularly in remote or difficult-to-access areas. Drones are known for their speed and agility, offering innovative solutions for logistics distribution.

However, solely relying on either trucks or drones presents certain limitations. Consequently, integrating both vehicles is anticipated to further improve distribution efficiency and service quality in rural logistics. This paper investigates the collaborative application of trucks and drones in rural logistics distribution. It will analyze the advantages and benefits of this partnership and the potential applications of drones within rural logistics, examining aspects such as technical limitations, application scenarios, distribution scope, and speed.

In terms of technological application and empirical research, this study will conduct simulation tests in rural settings to validate the proposed collaborative distribution model. Data and feedback from real-world operations will be gathered to evaluate its effectiveness in enhancing logistics distribution efficiency, reducing operating costs, and improving service quality.

The significance of this study is reflected in several key areas:

1. Enhance the efficiency and quality of logistics distribution. By integrating the collaborative operation of trucks and drones, we can optimize distribution routes, minimizing both time and costs while improving the accuracy and reliability of deliveries. This approach aims to address the growing logistics demands of rural residents.
2. Investigate new applications of drone technology in rural logistics. Drones represent an efficient and flexible solution that can effectively overcome the limitations of traditional delivery methods, particularly in mountainous areas or regions with challenging transportation access. They are poised to become a vital component of rural logistics in the future.
3. Offer technical support for the establishment of an intelligent logistics system. This study provides empirical research and theoretical insights by developing and validating a route planning model for truck-drone collaborative distribution. The goal is to advance logistics distribution services’ modernization and intelligent development.

## Literature review

Yan Qiong et al. believe that drones have attracted wide attention in logistics due to their advantages of fast speed, low cost, and close to a straight line. However, due to their limited flight mileage and load capacity, more and more scholars have begun to pay attention to the mixed distribution of trucks and drones to ensure their continuous operation^1^. Wu, GH established a hybrid integer programming model with minimum delivery time considering the drone energy consumption model^2^. Gao, JJ To address the complexity problem, we introduce an enhanced Gray Wolf Optimization algorithm (EGWO) that improves the initial solution through partitioning scanning and heuristic insertion algorithms^3^. Liang Shuang, aiming at the problems of high cost and low efficiency of logistics distribution in rural areas, studied the collaborative distribution of drones, trucks, and collection points to help the “rural revitalization” strategy^4^. Wang, DS suggests using trucks, truck drones, and standalone drones simultaneously to build more efficient truck-drone package delivery systems^5^. Xia, Y built an integer programming system to minimize the total cost of the drone’s weight-related costs, fixed vehicle costs, and flight distance costs^6^. Gou, MY presents an online delivery problem using trucks and some drones that comprise a hybrid truck-drone delivery collaborative system consisting of standalone and truck drones^7^. Bi, ZL believes that the “truck-drone” model solves both the range limitations of drones and the time wastage caused by trucks in the “last mile” delivery process^8^. Tong B. argues that the optimal delivery route problem for truck-drone transportation is defined as a traveling salesman with a drone problem (TSP-D), which has been extensively studied in previous literature. However, most existing studies have ignored waiting times for trucks at rendezvous points^9^. RF established a continuous traffic balance model in partial differential equations (PDEs) to describe the optimal drone route and truck-drone synchronization strategy for low-altitude air traffic congestion during large-scale steady-state operations^10^. To fully integrate truck-drone delivery in a contactless solution, Zhao L introduced the robust traveling salesman problem for drones, where drones make deliveries under uncertainty and return to a truck that is moving on its route^11^. Baldisseri, A. has evaluated last-mile delivery solutions’ environmental and economic sustainability for DRone-equipped electric trucks, comparing them to traditional logistics systems^12^. Luo, QZ solves the collaborative routing problem of truck-drone systems, where one truck collaborates with multiple drones to perform package delivery, and each customer can be served earlier or later than requested, given tolerances^13^. Xing, JH proposed a reliable truck-drone cooperative routing problem to realize dynamic synchronization between trucks and drones and proposed an integer programming model based on a spatiotemporal state network^14^. Zhang, LJ verified that truck-drone mode is more efficient than pure truck-delivery^15^.

Research conducted with literature reviews has identified several key hotspots in “drone-truck” collaborative delivery. Numerous studies have focused on optimizing the synergies between trucks and drones to address drones’ limitations regarding range and payload capacity while also leveraging trucks’ advantages for handling bulk shipments and emergency deliveries. This hybrid distribution model enhances logistics efficiency and resolves various practical challenges.

Secondly, the diversity of models and algorithms demonstrates adaptability to different distribution scenarios. For instance, the least squares method and simulated annealing algorithm are applied in epidemic spread models; the Mixed-Integer Linear Programming (MILP) model is utilized for solving truck-drone cooperative distribution; and the variable neighborhood search algorithm is used to optimize delivery routes. Each method has distinct characteristics, yet they share a common objective: improving distribution efficiency and resource utilization.

However, existing research has also highlighted some drawbacks of the hybrid distribution model, particularly concerning wait times between trucks and drones, synchronization issues, and their impact on overall system efficiency. To better address these challenges, future research should explore more effective ways to harness the advantages of both trucks and drones and optimize the dynamic coordination and real-time adjustment mechanisms between them.

The innovation of this paper is reflected in many aspects. First, through the collaborative delivery mode of drones and trucks, carbon emissions are effectively reduced, thus positively contributing to environmental protection. Secondly, given the complex terrain of rural China, the research carried out field simulation, demonstrating that drones can complete the distribution tasks that traditional trucks cannot achieve in mountainous areas, greatly improving logistics’ flexibility and response speed. In addition, the improved simulated annealing algorithm, k-means algorithm, and greedy algorithm are integrated in this paper to achieve higher efficiency and accuracy in logistics scheduling. The most important thing is that it innovatively proposes a new joint distribution collaboration mode, which subverts the time-consuming process of trucks waiting for drones in the traditional model, significantly reduces the time cost, and improves the overall distribution efficiency. These innovations provide new solutions for the logistics industry and open up new paths for sustainable development and the promotion of rural economies.

## Model construction

### Problem description

At present, the status quo of terminal distribution in rural areas is that trucks complete the delivery of all parcels within the distribution scope, while the actual scenario of the cluster-based collaborative distribution mode of trucks and drones is as follows: A truck carries four drones from the distribution center to each cluster, and collaborates with drones to complete the delivery of all parcels. The customer points served by trucks are those stopped by trucks, called truck service points or cluster points. The customer points served by drones are referred to as drone service points. A cluster is a collection of customer points consisting of a truck service point and several drone service points within the flight and load range of the drone near that point. The truck travels to the cluster point to complete the delivery of this customer point and operates the drone at this point to serve other customer points in the cluster. After completing one delivery service round, the drone returns to the cluster point for parcel loading and battery replacement. Then, he carries out the next delivery service until all customer points in the cluster have completed the service. After that, the truck carries the drone to the next cluster point to continue the service until all packages are completed and returned to the distribution center. Considering the use scenario of drones, it is stipulated that the distribution center must be a cluster point while considering the load limit of drones and the balance of parcel loading. It is stipulated that parcels exceeding the load range of drones must be distributed by trucks. Parcels not exceeding the load range of drones can be distributed by trucks or drones, and the optimization results determine the final distribution method. And the drone can carry multiple packages within the load and flight range.

To facilitate quantitative research, the problem is simplified, and the following assumptions are made:

(1) The demand for the distribution center is 0;

(2) All customer points must be met;

(3) the maximum range and maximum payload of the known drones;

(4) When the limit is met, the drones can serve multiple customer points in a single flight;

(5) The number of trucks and mileage limits are known, and the vehicle carries sufficient drone power;

(6) The drone cannot be released and recovered at will between different truck delivery points;

(7) The demand for each customer point is not greater than the maximum load of a single drone delivery.

### Symbol description

U: All node set, U=BUC;

B: Collection of truck distribution points, B={b|b=1,2,3,...,K};

C: Gather all customer points, C={c|c=1,2,3,....,M;

F={f|f=1,2,3,....,N} Assemble for drones;

K: Number of truck distribution points;

M: Number of customer points;

N: The number of drones;

m: The total number of trucks and vehicles available for dispatch;

u: Truck number;

L: The maximum range of the truck;

S: The total distance traveled by the truck;

CQ_C_: The demand of the customer point c;

F_m_: Cost per truck distance traveled;

W_1_: Drone delivery costs;

B_m_: Truck call cost;

d_hj_: Distance from distribution point h to distribution point j;

FQ_f_: Drones capacity;

FL_f_: Drone range;

BF_f_: Drones dispatch preparation cost;

W_f_: Drones loading and unloading cost;

F_f_: Drones unit distance transportation cost;

Z_hju_: The value is 0 or 1. 0 indicates that the truck is from the distribution point j to the distribution point h, and 1 indicates that the truck is from the distribution point h to the distribution point j.

I_hu_: The value is 0 or 1. 0 indicates that the items at the truck distribution point h are not delivered by the truck u, and 1 indicates that the items at the truck distribution point h are delivered by the truck u.

X_f_=1 Indicates that the drones f is put into use, otherwise X_f_=0;

y_cb_=1 Indicates that the demand point c is assigned to the point b service, otherwise y_cb_=0;

Z_ijf_=1 Indicates that the drone f flies from the delivery point i to the delivery point j, otherwise Z_ijf_=0.

### Model establishment

Once the trucks’ delivery points have been identified using the K-means algorithm, they depart from the distribution center. To enhance distribution efficiency and consider practical circumstances, this paper proposes utilizing trucks equipped with four drones, each assigned to specific delivery routes to various distribution points. Employing a greedy algorithm, the driving routes are optimized to minimize travel distance, which forms the basis for the objective function to model and solve.

The distribution center serves as the focal point for operations, where distribution services are initiated simultaneously toward the truck delivery points. The number of trucks available from the known distribution center is established, with the ultimate goal of minimizing travel distance as the primary objective. The objective function is constructed as follows:

1 $$\:min\:S\:=\:\:{\sum\:}_{h=0}^{k}\:\sum\:_{j=0}^{k}\:{\sum\:}_{u=0}^{k}{d}_{hj}\:{Z}_{hju}$$

2 $$\:s.t.{\sum\:}_{h=0}^{k}\:\left\{\begin{array}{c}=1,\:h=\text{1,2},\dots\:,k\\\:=m,\:\:\:\:\:\:\:\:\:\:\:\:\:h=0\end{array}\right.$$

3 $$\:{\sum\:}_{h=0}^{k}\:{\sum\:}_{j=0}^{k}\:{d}_{hj}\:{Z}_{hju}\le\:L$$

4 $$\:{\sum\:}_{h=1}^{k}\:{Z}_{hju}={l}_{ju},\:j=\text{0,1},2,\dots\:,k$$

5 $$\:{\sum\:}_{j=1}^{k}\:{Z}_{hju}={l}_{hu}$$

In the above distribution route optimization model, Eq. (1) is the objective function with the minimum total distance.

Formula (3) indicates that the total mileage of each truck distribution route must not exceed the mileage limit of trucks.

Formula (4) and Formula (5) eliminate the sub-loop between the truck distribution points.

With the goal of minimizing the delivery cost of drones, the following objective function is constructed:

6 $$\:min\:{W}_{1}\:=\:\sum\:_{f\in\:F}\left[{x}_{f}{BF}_{f}+\sum\:_{i\in\:U}\sum\:_{j\in\:U}{z}_{ijf}\left({d}_{ij}\text{*}{F}_{f}+{W}_{f}\right)\right]$$

7 $$\:s.t.\sum\:_{b\in\:B}{y}_{cb}=1,\forall\:c\in\:C$$

8 $$ \mathop \sum \limits_{{i \in U}} \mathop \sum \limits_{{j = C}} z_{{ijf}} CQ_{j}  \le FQ_{f} ,\forall f \in F $$

9 $$ \mathop \sum \limits_{{i \in U}} \mathop \sum \limits_{{j \in U}} z_{{ijf}} d_{{ij}}  \le FL_{f} ,\forall f \in F $$

10 $$ \mathop \sum \limits_{{i \in U}} z_{{ijf}}  - \mathop \sum \limits_{{i \in U}} z_{{ijf}}  = 0,\forall j \in U,f \in F $$

11 $$ \mathop \sum \limits_{{i \in U}} \mathop \sum \limits_{{f \in F}} z_{{ijf}}  = 1,\forall j \in C  $$

12 $$\:\sum\:_{i\in\:B}\sum\:_{f\in\:F}\sum\:_{j\in\:B}{z}_{ijf}=0,i\ne\:j$$

Objective function (6) represents the minimum cost of drones delivery, which is composed of preparation cost and actual transportation cost.

The constraint condition (7) indicates that any customer point can only correspond to one distribution point;

Formula (8) is to limit the maximum load of the drones;

Formula (9) is to limit the maximum mileage of the drones;

Formula (10) To ensure the drone delivery service route is closed loop;

Formula (11) To ensure that each customer point has and only one drone for delivery service;

Formula (12) indicates that the drone cannot be flown between truck delivery points.

Finally, the objective function of drones + truck joint delivery is constructed:

13 $$\:\text{m}\text{i}\text{n}\:\text{W}\:=\:\text{S}{\text{F}}_{\text{m}}\:+\:{\text{W}}_{1}\:+\:\text{m}{\text{B}}_{\text{m}}$$

Formula (13) indicates that the ultimate goal is the minimum total delivery cost of drones and trucks, which is composed of truck delivery cost, drone delivery cost, and truck scheduling cost.

## Algorithm design

### Determine the distribution point

We employ a two-stage clustering K-means algorithm on a dataset containing forty potential distribution points to determine the optimal locations for a truck distribution point and a central distribution point. The clustering algorithm operates: First, several objects are randomly selected from the dataset to serve as initial cluster centers. The remaining objects are then assigned to the cluster whose center they are most similar to, as measured by distance. After completing assignments, we calculate the new cluster center by finding the mean position of all objects within each cluster. This process is repeated until the standard measure function converges. Typically, the mean square error is used as the standard measurement function. The clusters formed are characterized by their compactness and separation from one another.

In the first stage of this study, we set the number of clusters, denoted as k, to divide the forty points based on their geographical locations and other relevant data. Each cluster will have a center of mass, the average position of all points within that cluster. In the second stage, after establishing k initial center points, the clustering algorithm is applied again to identify the secondary center points of these initial k centers, leading us to the final center points.

### Optimization of individual truck distribution routes based on a greedy algorithm

The basic idea of a greedy algorithm is, to begin with an initial solution to the problem and then iteratively make decisions that lead to a locally optimal solution at each step. At each stage, only one piece of data is considered, and its selection must satisfy the conditions for local optimization. If the next piece of data and the current partial optimal solution do not form a viable solution, that piece of data is not included in the partial solution. This process continues until all data has been evaluated or no further additions can be made.

A greedy algorithm generally follows these steps:

1. Build a mathematical model to describe the problem.
2. Divide the problem into several sub-problems.
3. Solve each sub-problem to obtain the local optimal solution.
4. Combine the local optimal solutions of the sub-problems to form a solution to the original problem.

The greedy algorithm is a straightforward and efficient design technique for certain optimization problems. Its defining feature is that it operates incrementally, making optimal choices based on the current situation without considering all possible scenarios. This approach saves considerable time that would otherwise be spent exhaustively searching for the best overall solution. The greedy algorithm employs a top-down, iterative method to make successive greedy choices, each simplifying the problem into a smaller sub-problem. By making these greedy selections at each step, one can find an optimal solution for the overall problem. However, while obtaining a local optimal solution at each step is essential, the resulting global solution may not always be the best possible. Hence, the greedy algorithm does not involve backtracking.

In this paper, the center point generated by the clustering algorithm from the previous section serves as the starting point for the greedy algorithm. The strategy then involves gradually expanding the route by selecting the nearest unvisited point from the current location as the next destination. This straightforward and effective selection method simplifies the path planning problem, often leading to a practical and reasonable

**Fig. 1:**
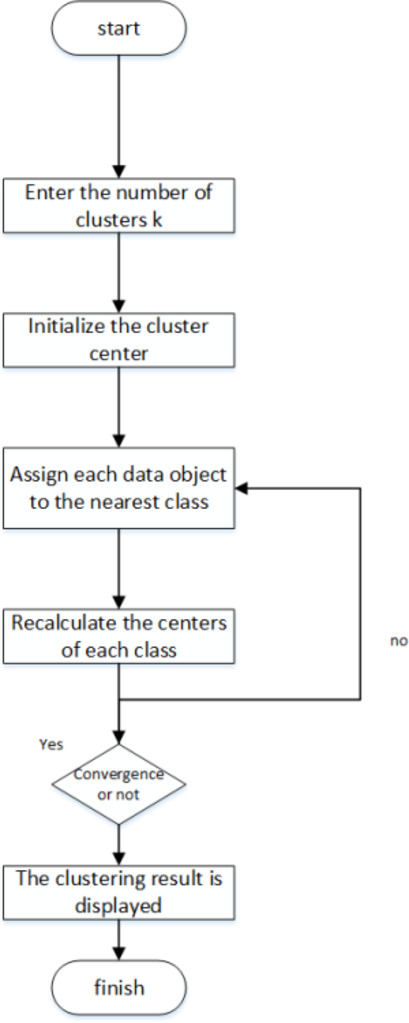
Clustering k-means algorithm.

solution. After each selection, the total length of the current route is updated, and the route is adjusted according to specific optimization goals. Figure 1 illustrates the algorithm.

### Determination of drone flight path based on simulated annealing algorithm

This paper selects the simulated annealing algorithm as the optimization tool because it can identify superior solutions within a complex search space. The simulated annealing algorithm effectively.

prevents the algorithm from becoming stuck in a local optimum by allowing for the acceptance of less favorable solutions. Over time, it progressively decreases the likelihood of accepting inferior solutions, guiding the process toward a global optimum.

**Fig. 2:**
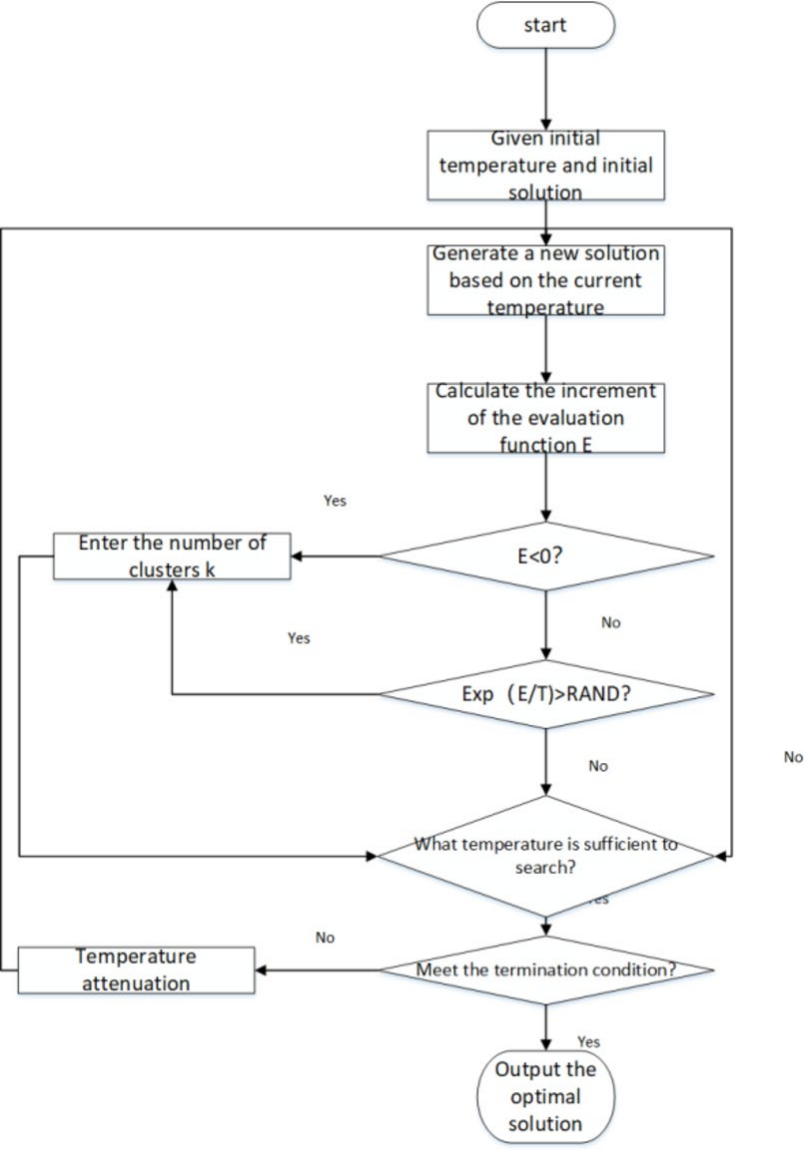
The idea of a simulated annealing algorithm.

In implementing the algorithm, an initial drone flight path is generated randomly. The iterative optimization process of the simulated annealing algorithm is then simulated, continually updating the current solution in the quest for a better flight path. During each iteration, the quality of each new solution is assessed by modifying the order of the flight path or the nodes within the selected path. Inferior solutions can be accepted based on a specific probability to avoid being trapped in local optima. Heuristic strategies are introduced to enhance the algorithm’s effectiveness, further improving the flight path’s quality and efficiency. Figure 2 illustrates the algorithm.

### Improving the idea of simulated annealing algorithm based on greedy algorithm

The performance of the simulated annealing algorithm largely depends on the quality of the initial solution. Randomly selecting the initial solution may cause the algorithm to become stuck around a suboptimal solution, making it more difficult to find the global optimal solution. To enhance the algorithm’s efficiency, a heuristic method can be employed to generate a better initial solution. This article proposes using a greedy algorithm to create that initial solution.

A greedy algorithm operates on the principle of making the most optimal choice at each step, hoping to build towards a globally optimal solution from these local optimizations. In the context of a path optimization problem, the greedy algorithm begins at a starting point and sequentially selects the nearest unvisited point until all points have been accessed. While this method does not guarantee finding the optimal solution, it often generates an initial path closer to the optimal one. This approach can help reduce search time in the simulated annealing process and increase the likelihood of identifying a global optimal solution.

Additionally, the performance of the simulated annealing algorithm is influenced by the temperature parameter and the number of iterations. The algorithm is more likely to accept worse solutions at higher temperatures, promoting exploration. Conversely, the algorithm becomes conservative at lower temperatures, accepting fewer inferior solutions. The cooling strategy, controlled by a parameter denoted as alpha, affects the rate at which the temperature decreases. A slower cooling rate (i.e., a smaller alpha) allows the algorithm more time to search around the optimal solution. Increasing the number of iterations means making more attempts at each temperature level, which enhances the chances of finding a globally optimal solution. By adjusting the iteration number, the algorithm can effectively avoid early stagnation in local optima, thereby improving convergence and the overall quality of the solution.

## Case studies and simulation experiments

### Case setting

The geographical environment of the Jinyun area in Lishui City, Zhejiang Province, is complex, including mountains, hills, and plains, which makes the design of distribution routes and strategies particularly important. By conducting empirical research in such an environment, drones’ delivery capability and efficiency under different terrain conditions can be better evaluated. Secondly, the rural economy of Jinyun is dominated by agriculture, with a rich variety of agricultural products, especially tea and fruit and other specialty products, which have a strong demand for efficient logistics distribution. This provides a real-world context for applying the “drone-truck” model, which can directly test its utility in the distribution of agricultural products. In addition, the rural population in Jinyun City is relatively concentrated. Although the distribution is more dispersed, there is a high customer density at key nodes, which provides a good coverage opportunity for the delivery of drones. In this case, evaluating how the joint distribution model can optimize the transportation path is possible. In addition, the Jinyun region has a high acceptance of new technologies, and the local government and farmers have an open attitude to the exploration of modern logistics means, which can provide a good basis for cooperation for the implementation of the research. Finally, the transportation network in the Jinyun area has gradually improved, providing convenience for the connection between drones and trucks. In this context, abundant data can be obtained through case calculation, which provides the practical basis and theoretical support for logistics model innovation in this region and other rural areas. These concrete reasons make Jinyun an ideal place to study the “drone-truck” joint distribution model.

**Fig. 3:**
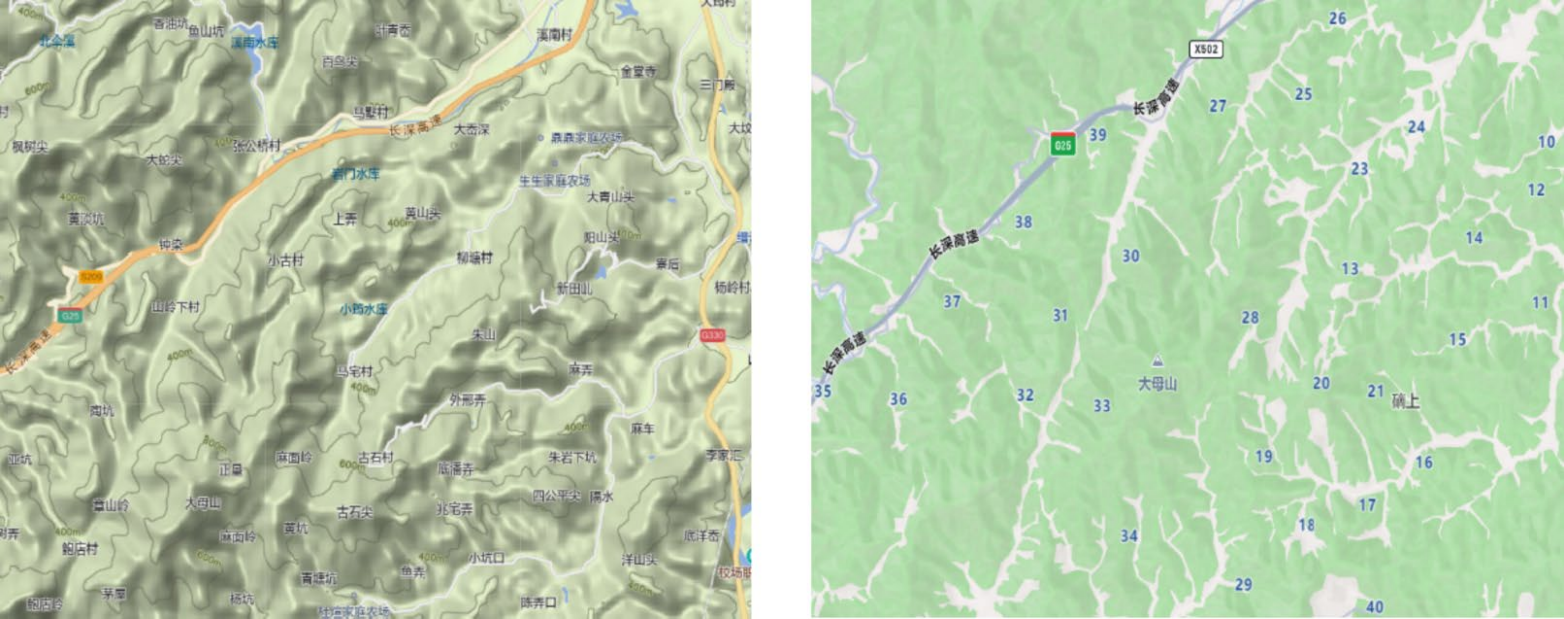
Village site selection diagram.

This paper examines a “drone-truck” joint distribution model based on 40 rural areas in the Jinyun region of Lishui City, Zhejiang Province. The study discusses a distribution system that leverages the cooperative operation of drones and trucks. The system functions as follows: First, a truck transports four drones to four designated secondary central points. Upon reaching these locations, each drone immediately begins its delivery task. After completing the deliveries, the truck retrieves the four drones along the same route and returns them to a primary central point for recharging. The latitude and longitude coordinates for each area were obtained from Baidu Maps, measured in degrees, and based on the geographical coordinate system. Figure 3shows the overall topography and site distribution of 40 villages.

Consider a scenario where a truck is equipped with four drones operating in the area, and customer demand is generated randomly. Table 1 presents the customer’s location and demand details, while Table 2 outlines the specifications of the vehicles and drones.

**Table 1:** Customer locations and requirements (Part).

village	Latitude(°)	Longitude(°)	Demanded(kg)
1	120.050967	28.688033	0.0
2	120.052692	28.678654	3.4
3	120.051398	28.668766	2.8
4	120.054417	28.650636	3.7
5	120.051254	28.639478	1.0
6	120.044787	28.629079	4.9
7	120.038175	28.657609	3.6
8	120.040044	28.6481	0.2
…	…	…	…
31	119.937709	28.649368	2.8
32	119.931385	28.639224	4.7
33	119.945039	28.637829	0.9
34	119.949782	28.621089	1.2
35	119.896027	28.639731	4.8
36	119.90925	28.638717	0.3
37	119.918593	28.651143	2.1
38	119.931097	28.661286	0.3
39	119.94432	28.672569	0.1
40	119.992954	28.612017	0.7

**Table 2:** Related parameters of trucks and drones.

Truck	Dispatch preparation cost $\:{B}_{m}$	100¥/car
	Unit driving cost $\:{F}_{m}$	10¥/km
Drone	Load $\:{FQ}_{f}$	15 kg
	Maximum range $\:{FL}_{f}$	35 km
	Maximum round-trip linear distance $\:{H}_{max}$	30 m
	Dispatch preparation cost $\:{BF}_{f}$	30¥/each
	Handling cost $\:{W}_{f}$	2¥/time
	Transportation cost per unit distance $\:{F}_{f}$	5¥/km

### Determine the truck delivery point

To clarify the results of customer point clustering, this paper first establishes the number of clusters as k = 4. By applying the k-means clustering algorithm, all customer points are categorized into four distinct areas. The fundamental principle of k-means is to minimize the objective function:

14 $$ Z = \sum\limits_{{i = 1}} {\sum\limits_{{x \in C_{i} }} {\left\| {x - U_{i} } \right\|} } ^{2}   $$

In the above formula, $\:Z\:$represents the total square error, $\:{C}_{i}$ represents the $\:i$-th cluster, $\:x$ is the data point, and $\:{U}_{i}$ is the center of mass of the $\:i$-th cluster. Finally, four distribution centers and their corresponding distribution areas are formed. Customer points in each delivery area are within the range of drone delivery, and there are no abnormal points in all customer points. The coordinates of these distribution centers are A1[120.0404,28.6559], A2[119.9313,28.6468], A3[119.9908,28.6666] and A4[119.9842,28.6295].

**Fig. 4:**
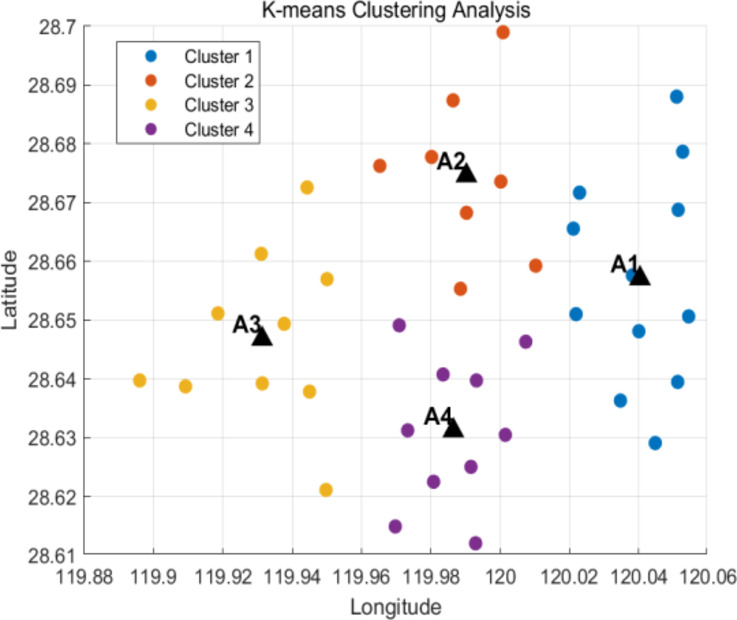
K-means cluster analysis results (Stage 1).

As illustrated in Fig. 4, the final four distribution regions are linked to their respective distribution centers, where customer points in each region are effectively allocated to the nearest distribution center. This allocation ensures an efficient distribution process and guarantees that each customer point falls within the drone’s delivery range, thus preventing potential distribution problems caused by an excessive delivery distance.

The results demonstrate that by establishing a clustering scheme with k = 4, this paper effectively assigns customer points to different distribution areas, facilitating a smooth delivery process. The drones’ delivery range can cover all customer points, optimizing the distribution service, enhancing distribution efficiency, and mitigating challenges posed by the uneven distribution of customer points. This method has been validated not only theoretically but also through practical application.

In the initial phase, the paper divides all customer points into four distinct distribution areas by setting the number of clusters to k = 4 and identifies four preliminary distribution centers. Table 3 presents the results of the cluster analysis. To further refine the location of each truck distribution center, a second stage of analysis is conducted, employing a clustering algorithm based on center point updates.

During this stage, the clustering algorithm is applied again, focusing on the center point update method to confirm the final location of the distribution centers. This step involves recalculating the mean of all customer points within each region to reposition the center point accurately. This approach ensures that the central point better reflects the actual distribution of customer points, thereby enhancing overall distribution efficiency.

**Fig. 5:**
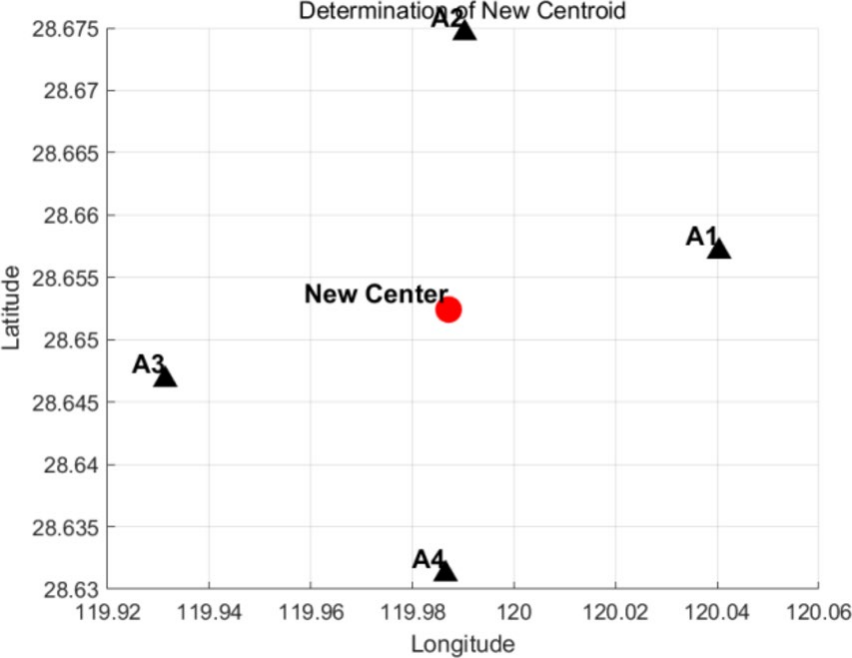
K-means cluster analysis results (Stage 2).

After thorough calculations and analysis, the final coordinates for the distribution center are determined to be [119.9879, 28.6510]. As depicted in Fig. 5, this new coordinate results from optimizing the initially determined center point in the previous stage. The updated central point improves the balance of customer distribution across the various regions, optimizes delivery routes, and minimizes potential discrepancies in the distribution process.

**Table 3:** Cluster analysis of distribution areas.

Distribution area	Village point
Distribution area A1	30,31,32,33 35,36,37,38,39
Distribution area A2	1,2,3,4,5,6,7,8,9,10,11,12
Distribution area A3	13,14,15,22,23,24,25,26,27,40
Distribution area A4	16,17,18,19,20,21,28,29,34

### Greedy algorithm optimizes truck delivery routes

This paper employs a greedy algorithm to determine the optimal distribution path within the distribution area. First, the coordinate data for all customer points and the demand for each point are extracted from Table 1. This data is then inputted into MATLAB for further analysis and calculation.

This study first needs to make the necessary conversions to convert the longitude and latitude coordinates into actual kilometers. This step is crucial for ensuring the accuracy of distance calculations, as the units of latitude and longitude are typically expressed in degrees. Using these units directly would yield unrealistic distance measurements. In MATLAB, each customer point’s latitude and longitude coordinates are converted into a plane coordinate system measured in kilometers. The specific steps for this conversion are as follows:

Step 1: Input latitude and longitude data into MATLAB. This data typically includes the coordinates of each point, which may be stored in an array or matrix.

Step 2: Coordinate conversion is necessary for latitude and longitude coordinates, expressed in degrees. To use most geographical formulas, these degrees must be converted to radians. This conversion is typically performed by multiplying the degree value by π/180.

Step 3: Using the Haversine formula to calculate the distance between two points on the Earth’s surface. This formula accounts for the curvature of the Earth and is suitable for measuring distances on a sphere. It involves determining the differences in latitude and longitude between the two points and then using these differences in the formula to calculate the distance.

Step 4: Calculated distances are typically expressed in units of Earth’s radius. The resulting distance is given in kilometers when using the Earth’s radius (6,371 km).

Step 5: Result output. Output the calculated distance, usually in kilometers.

After the coordinate conversion is completed, a greedy algorithm plans the path. The core idea of the greedy algorithm is to choose the current optimal path each time to finally get a global approximate optimal solution. In this analysis, the greedy algorithm of this paper selects the nearest customer point each time as the next distribution target so as to gradually build the shortest distribution path.

**Fig. 6:**
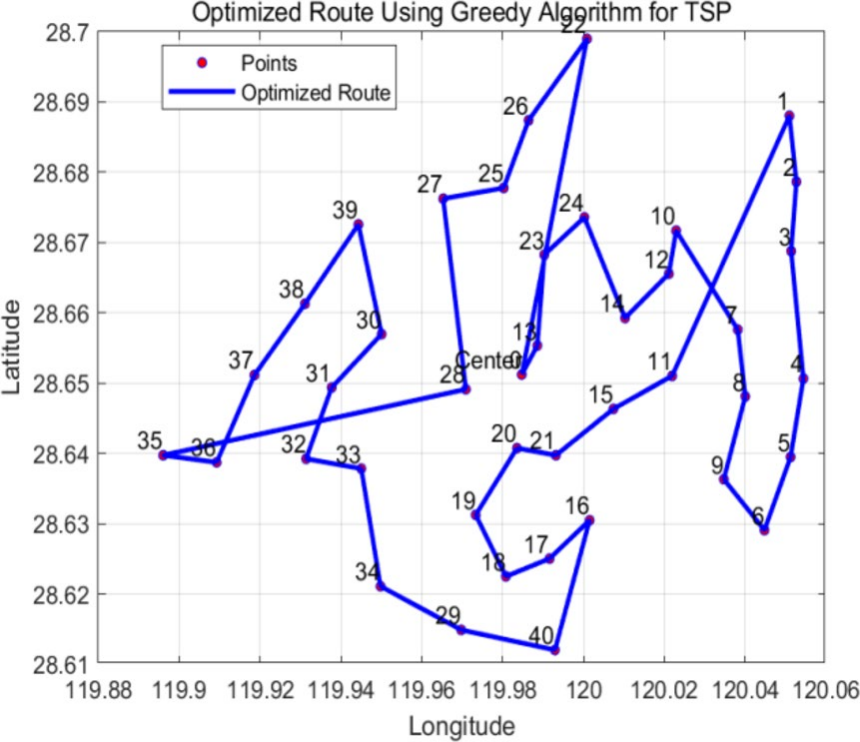
Greedy algorithm path for truck distribution alone.

Figure 6 illustrates the route diagram, detailing the connections between each customer point and the final calculated delivery path. This visualization allows for an intuitive understanding of the results at each step and the overall distribution path.

### Determine the route of the drone

First, input the coordinates and demand data of customer locations from Table 3 for each distribution area into MATLAB. This includes the longitude and latitude coordinates for each customer and their respective demand, ensuring that the latitude and longitude are converted into a suitable coordinate system. This problem aims to optimize the drone’s flight path using a simulated annealing algorithm to minimize the total delivery distance or cost.

The process takes into account various constraints, such as the drone’s maximum flight range, service time, and demand in each distribution area. During the initialization phase, an initial flight path is generated, which can be the starting solution derived from a greedy algorithm or a randomly generated path. The initial temperature is set to 1000 to influence the probability of the algorithm accepting less optimal solutions, while the temperature drop coefficient is set to 0.95 to control the rate of temperature decline.

A new solution is generated in the simulated annealing process by making local perturbations to the current path. This can involve exchanging the positions of two points in the path. These perturbations form the neighborhood structure of the algorithm. A distance calculation function is employed to evaluate the total distance and the cost of the current and new paths. The decision to accept the new solution is based on the Metropolis criterion: the algorithm may update the current path even if the new solution has a longer path length, or it may accept a longer path with a certain probability, which is dictated by the current temperature.

As the algorithm progresses, the temperature is updated according to the defined drop coefficient, gradually reducing the likelihood of accepting suboptimal solutions. The termination conditions specify that the algorithm will stop when the temperature falls to a predetermined minimum value or after reaching a maximum number of iterations. Once concluded, the algorithm outputs the optimal solution, including the best path discovered through simulated annealing and its associated total distance or cost.

**Fig. 7:**
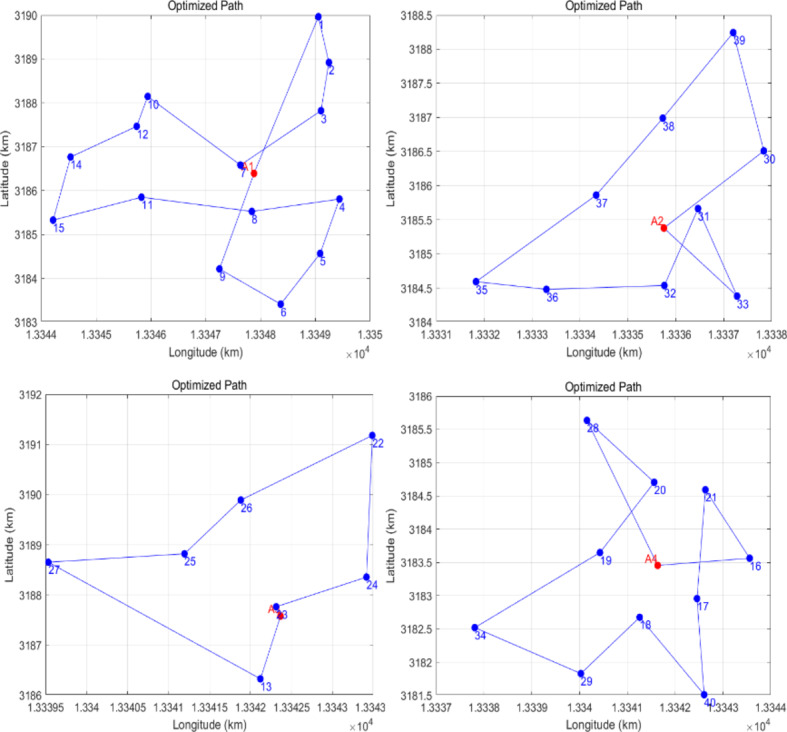
Path of drones simulated annealing algorithm.

Finally, the optimal path and the customer points in each distribution area are visualized, which aids in analyzing the drone flight paths and the layout of the distribution areas. This helps optimize the drone’s actual operational strategy. These steps demonstrate how the simulated annealing algorithm can effectively determine the flight paths of drones and enhance distribution efficiency. The specific route is illustrated in Fig. 7.

To determine the drones’ flight path more effectively, we employ an enhanced simulated annealing algorithm combined with a greedy algorithm to optimize the initial solution. First, we input the coordinates of customer locations and demand data from each distribution area, as shown in Table 3, into MATLAB. This data includes the latitude and longitude of each customer along with their requirements, which must be converted into kilometer coordinates for distance calculations. We begin by setting the initial temperature to 1000 and the temperature drop coefficient to 0.95 while defining the number of iterations for the simulated annealing to 1000 to ensure thorough exploration of the solution space. The improved simulated annealing algorithm starts by generating a good initial solution using the greedy algorithm. The steps of the greedy algorithm involve beginning from a starting point and selecting the closest unvisited point to the current location at each step, gradually constructing a more efficient path. The initial solution generated in this way is typically closer to the optimal solution than a randomly generated one, thereby reducing the convergence time of the simulated annealing algorithm.

**Fig. 8:**
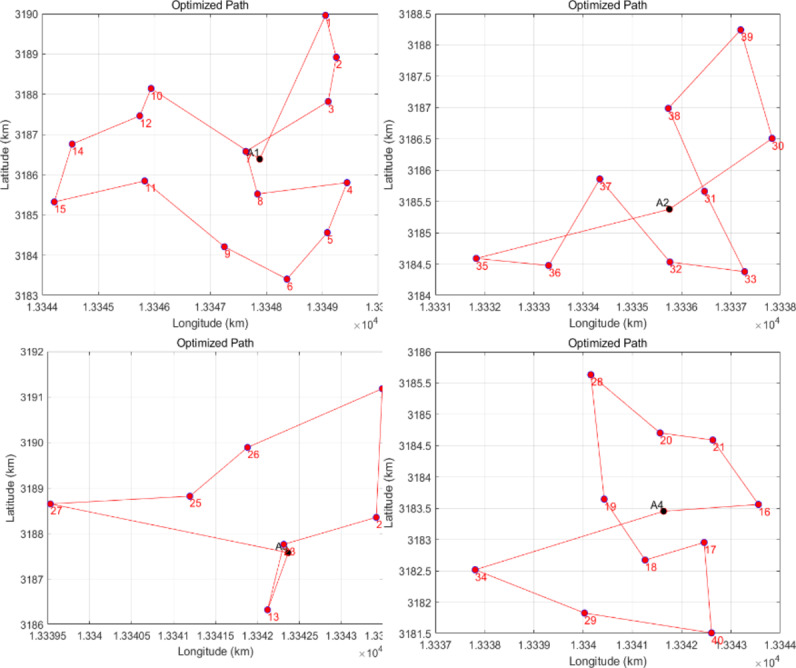
Drones improved the simulated annealing algorithm path.

Finally, we present the optimal path identified by the simulated annealing algorithm and its total distance or cost. By illustrating the route diagram for each distribution area (as shown in Fig. 8), we can visually depict the drones’ optimized flight routes and the layout of each distribution area. This visualization aids in analyzing and enhancing the drones’ distribution strategy.

The improved simulated annealing algorithm leverages a high-quality initial solution generated by a greedy algorithm, significantly boosting both the efficiency and quality of the results.

The improved simulated annealing algorithm leverages a high-quality initial solution generated by a greedy algorithm, significantly boosting both the efficiency and quality of the results. Table 4 summarizes the paths and distances for each route. A comparative analysis of the drone flight path optimization experiment within the distribution area reveals that the mileage of the improved simulated annealing algorithm is reduced by 7.58 km, resulting in an enhancement of 11.68% compared to the unimproved version of the algorithm.

**Table 4:** Detailed paths of each route.

route	Simulated annealing algorithm-specific path	Distance (km)	Improve the specific path of the simulated annealing algorithm	Distance (km)
L1	A1-1-2-3-7-10-12-14-15-11-8-4-5-6-9-A1	19.36	A1-1-2-3-7-8-4-5-6-9-11-15-14-12-10-A1	18.26
L2	A2-30-39-38-37-35-36-32-31-33-A2	16.80	A2-31-30-39-38-33-32-37-36-35-A2	13.49
L3	A3-23-24-22-26-25-27-13-A3	12.56	A3-13-23-24-22-26-25-27-A3	10.53
L4	A4-16-21-17-40-18-29-34-19-20-28-A4	16.18	A4-16-21-20-28-19-18-17-40-29-34-A4	15.04
Total distance	/	64.9	/	57.32

## Discussion and outlook

### Result analysis

**Fig. 9:**
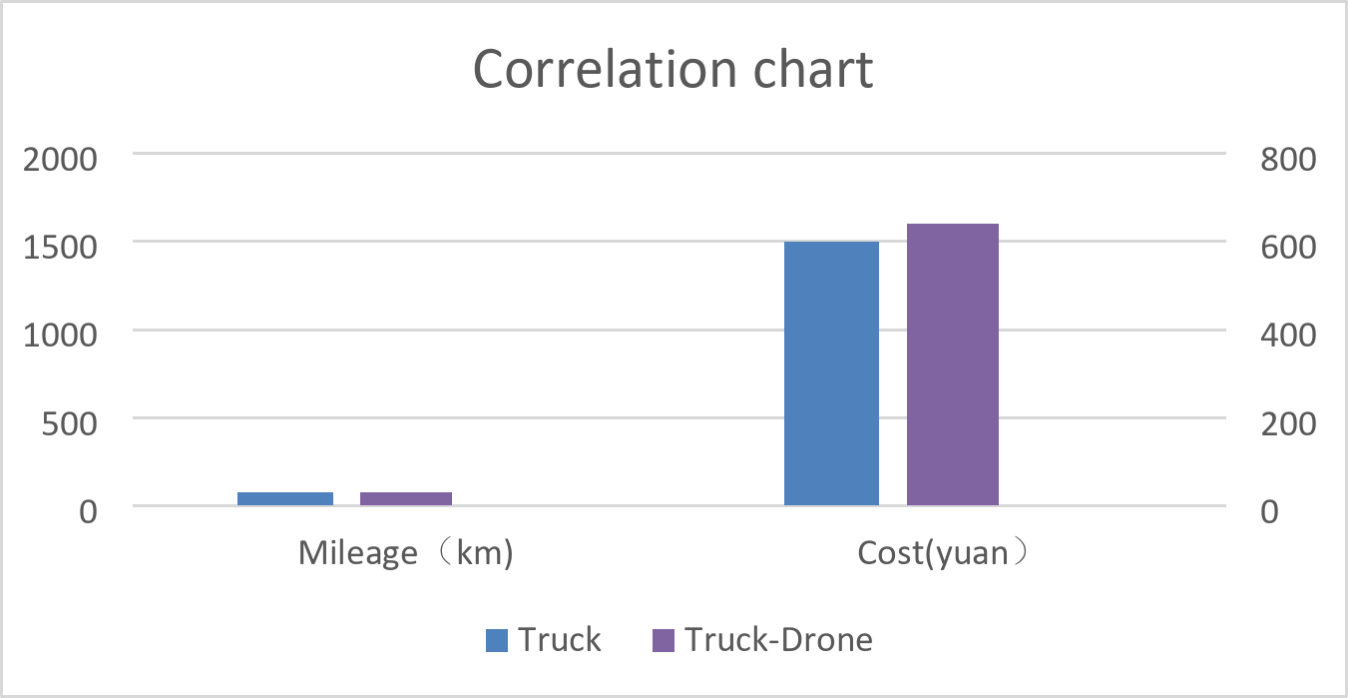
Comparison of process costs between the two methods.

The simulated annealing algorithm was employed to determine that the total mileage for truck-only distribution is 75.06 km, resulting in a cost of 1501.2 yuan. In contrast, the total mileage for the “truck-drone” joint distribution mode is 89.36 km, costing 927.4 yuan. This indicates a reduction in distribution costs of 573.8 yuan, which is approximately 38.22% lower than the truck-only distribution costs. Refer to Fig. 9 for further details.

### Research conclusions

The drone-truck collaborative distribution model discussed in this study significantly contributes to sustainable logistics and rural economic development. By optimizing logistics routes, this model effectively reduces carbon emissions during transportation, minimizes environmental impact, and supports sustainable development goals. The flexible delivery capabilities of drones enhance the market competitiveness of agricultural products by ensuring the rapid delivery of fresh goods to consumers. This reduces losses associated with long-distance transport and increases farmers’ incomes. Utilizing drone technology to improve rural logistics services can attract more investment and promote the transformation and upgrading of regional economies. Furthermore, this study offers a scientific basis for policymakers to encourage and support the adoption of drone logistics technology, facilitating the integration of green logistics with rural revitalization strategies. Overall, this model enhances logistics efficiency and provides crucial support for sustainable development and the coordinated advancement of the rural economy.

In this paper, we determine truck distribution points using the K-means clustering algorithm. We optimize the driving paths of trucks with a greedy algorithm and establish the collaborative delivery routes of a “truck-drone” system using a simulated annealing algorithm. The K-means clustering algorithm effectively identifies high-demand distribution points and optimizes resource allocation through efficient region partitioning. This algorithm relies on the distances between data points and iteratively adjusts the cluster centers to ensure that the distribution tasks align with the actual demand. As a result, it reduces the drone’s flight paths and transportation times, significantly enhancing overall distribution efficiency.

The greedy algorithm ensures real-time responses and efficient resource utilization by prioritizing the nearest tasks when calculating the delivery paths for individual trucks. This strategy minimizes wait times and optimizes logistics processes, allowing smoother collaboration between trucks and drones. Moreover, incorporating a greedy strategy, the improved simulated annealing algorithm enhances global search capabilities. It approaches a globally optimal solution quickly within complex solution spaces. This algorithm dynamically adjusts the temperature, enabling it to accept new solutions even at low temperatures during the iterative process. It avoids local optima and improves path selection flexibility and accuracy.

Together, the combination of these three algorithms significantly enhances distribution system performance in terms of efficiency and flexibility. The following conclusions have been drawn:

(1) The research indicates that the “truck-drone” collaborative path planning strategy significantly reduces logistics costs and improves economic benefits.

(2) The application of the improved simulated annealing algorithm effectively optimizes the “truck-drone” joint distribution model. Although total mileage may increase, overall costs are notably reduced, demonstrating the algorithm’s ability to find better solutions in complex environments. This model not only shortens transportation time but also more efficiently meets the logistics needs of rural areas. Additionally, using drones for delivery reduces the environmental impact, contributing to the sustainability of logistics transportation.

However, several limitations regarding drone applications in rural logistics distribution must be considered. First, weather conditions can significantly affect the flight safety of drones, particularly in rural areas. Safety issues also cannot be overlooked, as drones may collide with people or objects during flight and could experience technical failures. Furthermore, local regulatory restrictions can impact drone usage, especially near highways. Finally, economic factors, including maintenance and operating costs, will influence drone viability in rural delivery.

These considerations help comprehensively evaluate the practical feasibility and future development directions for drones in rural logistics distribution.

In future research, we can delve further into optimizing and scaling the truck-drone collaborative path planning model to address rural logistics delivery services’ complex challenges and continuous improvement needs. Specifically, more refined routing algorithms can be researched and developed to reduce transportation costs and enhance service efficiency. In addition to the greedy and simulated annealing algorithms already in use, we can incorporate advanced techniques such as deep learning or reinforcement learning to enable smarter decision-making and path optimization. Additionally, future studies could integrate real-time traffic data and predictive models, considering the road congestion index to dynamically adjust the paths and itineraries for trucks and drones. This approach will enable more effective responses to emergencies and traffic changes, thereby improving the stability and punctuality of distributions. To address the high computational complexity associated with the simulated annealing algorithm, new technologies such as parallel computing, distributed algorithms, or quantum computing can be explored to expedite the path optimization process and reduce computational costs.

In implementing the truck-drone joint distribution model, it is essential to thoroughly study and address the challenges related to the construction of technical facilities, operation management, and compliance with laws and regulations. This is particularly important in rural areas, where customized solutions are needed to fit local environmental and economic conditions. To continually assess and manage the risks associated with changes in logistics costs, future research should focus on designing and implementing long-term monitoring and evaluation mechanisms. By combining actual operational data, strategies can be adjusted promptly, and models can be optimized to ensure stable long-term operations and effective cost control.

## Electronic Supplementary Material

Below is the link to the electronic supplementary material.

Supplementary Material 1

## Data Availability

Data is provided within the manuscript or supplementary information files.
